# Investigation of enhanced intracellular delivery of nanomaterials modified with novel cell-penetrating zwitterionic peptide-lipid derivatives

**DOI:** 10.1080/10717544.2023.2191891

**Published:** 2023-03-25

**Authors:** Yuri Sugimoto, Tadaharu Suga, Mizuki Umino, Asako Yamayoshi, Hidefumi Mukai, Shigeru Kawakami

**Affiliations:** a Department of Pharmaceutical Informatics, Graduate School of Biomedical Sciences, Nagasaki University, Nagasaki, Japan; b Department of Chemistry of Functional Molecules, Graduate School of Biomedical Sciences, Nagasaki University, Nagasaki, Japan; c Laboratory for Molecular Delivery and Imaging Technology, RIKEN Center for Biosystems Dynamics Research, Kobe, Japan

**Keywords:** Liposomes, lipid nanoparticles, extracellular vesicles, cell-penetrating peptides, polyethylene glycol, microfluidics

## Abstract

Functionalized drug delivery systems have been investigated to improve the targetability and intracellular translocation of therapeutic drugs. We developed high functionality and quality lipids that met unique requirements, focusing on the quality of functional lipids for the preparation of targeted nanoparticles using microfluidic devices. While searching for a lipid with high solubility and dispersibility in solvents, which is one of the requirements, we noted that KK-(EK)_4_-lipid imparts nonspecific cellular association to polyethylene glycol (PEG)-modified (PEGylated) liposomes, such as cell-penetrating peptides (CPPs). We investigated whether KK-(EK)_4_-lipid, which has a near-neutral charge, is a novel CPP-modified lipid that enhances the intracellular translocation of nanoparticles. However, the cellular association mechanism of KK-(EK)_4_-lipid is unknown. Therefore, we synthesized (EK)_n_-lipid derivatives based on the sequence of KK-(EK)_4_-lipid and determined the sequence sites involved in cellular association. In addition, KK-(EK)_4_-lipid was applied to extracellular vesicles (EVs) and mRNA encapsulated lipid nanoparticles (mRNA-LNPs). KK-(EK)_4_-lipid-modified EVs and mRNA-LNPs showed higher cellular association and in vitro protein expression, respectively, compared to unmodified ones. We elucidated KK-(EK)_4_-lipid to have potential for applicability in the intracellular delivery of liposomes, EVs, and mRNA-LNPs.

## 1. Introduction

Lipid-based nanoparticles, such as liposomes and lipid nanoparticles (LNPs), have been used as carriers for drug and gene delivery (Furneri et al., 2000; Landen et al., 2005; Kulkarni et al., 2018; Ndeupen et al., 2021; Mukai et al., 2022). Furthermore, exosomes, which are cell-derived vesicles that have a structure similar to that of liposomes and contain nucleic acids and proteins, have been investigated as novel drug delivery systems (DDS) (Chung et al., 2016; Kim et al., 2016; Lu et al., 2018; Rayamajhi et al., 2019; Zheng et al., 2019). The functionalization of DDS carriers has been investigated for the efficient delivery of therapeutic drugs and has been reported to improve targeting and intracellular translocation by modifying functional lipids with molecules, such as antibodies, small molecules, and peptides (Song et al., 2009; Kibria et al., 2011; Lu et al., 2018; Moghimipour et al., 2018). The quality of functional lipids, such as molecular weight distribution, dispersibility in water, and solubility in solvents, is important in terms of the production and quality control of nanoparticles.

In recent years, nanoparticle preparation methods using microfluidic devices have attracted increased attention as novel preparation methods, as they are used in methods for manufacturing LNP formulations, such as Onpattro®. Mixing between organic solvents and buffers in microchannels promotes the self-assembly of nanoparticles, and high-speed and strictly controlled mixing enables the preparation of uniform and highly reproducible nanoparticles (Zhigaltsev et al., 2012; Maeki et al., 2017).

In this way, the quality of functional lipids and the manufacturing method of nanoparticles play a crucial role in the clinical use of nanoparticles. Thus, we focused on the quality of functional lipids and reported targeted PEGylated liposomes using high functionality and quality (HFQ) lipids (Suga et al., 2017, 2018, 2019; Kato et al., 2022). HFQ lipids were designed and synthesized to meet unique requirements regarding lipid components, dispersibility, and solubility in solvents for preparing highly reproducible functionalized nanoparticles using microfluidic devices. Ligand-[serine and glycine (SG)]_5_-lipid is a series of HFQ lipids composed of a targeting moiety, flexible peptide spacer (non-α-helix structure), and lipid tails. However, ligand-(SG)_5_-lipid is not applicable to the one-step formulation of nanoparticles using a microfluidic device because of its lower solubility in ethanol. Therefore, we designed a new HFQ lipid structure to improve ethanol solubility by substituting the (SG)_5_ spacer with other peptide spacers. One of the peptides we tested was an EK repeat peptide composed of negatively charged glutamic acid (E) and positively charged lysine (K). It has been reported that EK repeat peptides, such as PEG, have anti-fouling effects and prolong the blood circulation times of nanoparticles (Nowinski et al., 2012; Zhao et al., 2018).

Cell-penetrating peptides (CPPs) have been used for the intracellular delivery of proteins, nucleic acids, and nanoparticles (Schwarze et al., 1999; Fretz et al., 2004; Kim et al., 2006; Asai et al., 2014; Nakase et al., 2017). CPPs are typically arginine-rich peptides that contain six or more arginine residues in their sequence (Futaki et al., 2001). Consequently, unintended interactions between cationic CPPs and anionic materials, such as nucleic acids and exosomes, are often difficult to prepare. In addition, excess cationic material on the nanoparticles would cause cellular cytotoxicity and platelet aggregation. Therefore, a new material to improve the cellular association with fewer cationic net charges is needed.

The process of systemic evaluation of various lipids with EK repeats revealed that the cellular association of KK-(EK)_4_-lipid-modified nanoparticles was remarkable, despite the absence of a targeting ligand. Considering these observations, we hypothesized that KK-(EK)_4_-lipid, which provides cellular association properties to PEGylated liposomes despite their near-neutral charge, can be used as a novel CPP-modified lipid to improve the intracellular translocation of nanoparticles. However, the detailed cellular association mechanism of KK-(EK)_4_-lipid is still unknown. Therefore, to determine the effect of amino acids in the sequence of KK-(EK)_4_-lipid, we synthesized (EK)_n_-lipid derivatives and prepared liposomes modified with the derivatives. Next, we compared their cellular associations. Furthermore, we evaluated the intracellular localization and endocytic pathway of the liposomes to investigate the intracellular distribution of KK-(EK)_4_-lipid-modified liposomes. In addition, because KK-(EK)_4_-lipid has high dispersibility and solubility in both water and ethanol solutions, KK-(EK)_4_-lipid was applied to extracellular vesicles (EVs) and mRNA-encapsulated LNPs (mRNA-LNPs) to determine the usefulness of KK-(EK)_4_-lipid for the intracellular delivery of nanocarriers other than liposomes.

## 2. Materials and methods

### 2.1. Materials

Fmoc-amino acids and Rink Amide AM resin were obtained from Merck (Darmstadt, Germany). NBD-DOPE, rhodamine-DOPE, and 1,2-distearoyl-sn-glycero-3-phosphocholine (DSPC) were obtained from Avanti Polar Lipids (Alabaster, AL). 1,2-Distearoyl-sn-glycero-3-phosphoethanolamine- N-[methoxy (polyethylene glycol)-2000] (mPEG2000-DSPE) and DLin-MC3-DMA were purchased from NOF (Tokyo, Japan) and MedChemExpress (Monmouth Junction, NJ, USA), respectively. All other chemicals were reagent-grade, commercially obtained products.

### 2.2. Synthesis of (EK)_n_-lipid derivatives

(EK)_n_-lipid derivatives were synthesized by solid-phase peptide synthesis as described previously (Suga et al., 2017, 2018). The compounds were purified and assessed using RP-HPLC and MALDI-TOF-MS.

### 2.3. Preparation of PEGylated liposomes

The lipid composition of the liposomes was DSPC/Cholesterol/mPEG2000-DSPE [60:35:5 (molar ratio)], and each lipid was dissolved in methanol. For fluorescence labeling, 0.5 mol% rhodamine-DOPE or NBD-DOPE was used. After evaporation and desiccation, the lipid film was hydrated in sterilized water for 10 min and sonicated for 3 min at 65 °C.

### 2.4. Modification of KK-(EK)_4_-lipid into PEGylated liposomes

In the preparation using the bulk mixing post-insertion method, KK-(EK)_4_-lipid micelles (6 mol%) were incubated with PEGylated liposomes for 1 h at 60 °C. In the preparation using the microfluidic post-insertion method, the heat block of NanoAssemblr Benchtop (Precision NanoSystems Inc., Vancouver, BC, Canada) was set at 60 °C, and PEGylated liposomes and 6 mol% KK-(EK)_4_-lipid micelles were mixed at a total flow rate (TFR) of 1 mL and a flow rate ratio (FRR) of 1:1 onto the microfluidic chip as previously described (Sugimoto et al., 2022). After reaching room temperature, the isotonic properties of the liposomes were adjusted by the addition of 10 × phosphate buffered saline (PBS, pH 7.4), and the samples were filtered through a 0.45-μm filter. The physicochemical properties of the samples were measured using a Zetasizer Nano ZS and a Zetasizer Pro (Malvern Instruments Ltd., Worcestershire, UK).

### 2.5. Modification of KK-(EK)_4_-lipid into EVs

Bovine milk exosomes (COSMO Bio, Co., Ltd., Tokyo, Japan), which are commercially available exosomes, were used as the EVs. For fluorescence labeling, using previously described method (Morales-Kastresana et al., 2017), EVs were stained with CFDA-SE (Invitrogen, USA), commonly referred to as CFSE. The protein concentration of EVs was determined using a microBCA Protein Assay Reagent Kit (Thermo Scientific). KK-(EK)_4_/EVs were prepared by adding an equal amount of KK-(EK)_4_-lipid micelles and EVs as protein. In the preparation using bulk mixing post-insertion method, KK-(EK)_4_-lipid micelles and EVs were incubated for 1 h at 37 °C. In the preparation using microfluidic post-insertion method, the heat block of NanoAssemblr Benchtop was set at 37 °C, and EVs and KK-(EK)_4_-lipid micelles were mixed at a TFR of 1 mL and a FRR of 1:1 onto the microfluidic chip. The physicochemical properties of the samples were measured using a Zetasizer Nano ZS or a Zetasizer Pro (Malvern Instruments Ltd., Worcestershire, UK).

### 2.6. Modification of KK-(EK)_4_-lipid into mRNA-LNPs

mRNA-LNPs were prepared using a NanoAssemblr Benchtop as previously described (Kamiya et al., 2022; Ogawa et al., 2022). The lipid composition of the LNPs was Dlin-MC3-DMA/DSPC/Cholesterol/DMG-PEG2000/KK-(EK)_4_-lipid [50:10:38.5:1.5:1 (molar ratio)], and each lipid was dissolved in ethanol. In addition, the mRNA was dissolved in 50 mM citrate buffer (pH 3). The lipid and mRNA solutions were mixed on the microfluidic chip at a TFR of 4 mL and a FRR of 1:3. The obtained solution was dialyzed against PBS (pH 7.4) to remove ethanol and then concentrated via ultrafiltration. The physicochemical properties of the samples were measured using a Zetasizer Nano ZS and a Zetasizer Pro (Malvern Instruments Ltd., Worcestershire, UK).

### 2.7. Cell culture

A549 and MIA Paca-2 cells were purchased from the RIKEN Cell Bank (Tsukuba, Japan). Colon-26 and NIH3T3 cells were obtained from the Cell Resource Center for Biomedical Research (Tohoku University, Sendai, Japan) and ECACC (Porton Down, Salisbury, UK), respectively. The cells were cultured in medium containing 10% heat-inactivated fetal bovine serum (FBS) (Bovogen, East Keilor, VIC, Australia), 100 U/mL penicillin, and 100 μg/mL streptomycin (Wako, Osaka, Japan) in an atmosphere of 5% CO_2_ at 37 °C.

### 2.8. Cellular association analysis

The cells were seeded (2.5 × 10^4^ cells/cm^2^) and incubated for 24 h. Rhodamine-labelled liposomes (25 μM) or CFSE-labelled EVs (5 µg/mL) in serum-free medium were added to the cells. After 3 h, cells were washed with PBS and collected. The cells were analyzed using a BD LSR Fortessa flow cytometry (BD Biosciences, San Jose, CA, USA).

### 2.9. Confocal laser scanning microscopy

The cells were seeded (2.5 × 10^4^ cells/cm^2^) and incubated for 24 h. NBD-labelled liposomes (25 μM) in serum-free medium were added to the cells and incubated for 3 h. After washing the cells with PBS, they were incubated with LysoTracker Red DND-99 (50 nM) for 1 h and then washed with PBS. The cells were stained with Hoechst 33342 (5 µg/mL) for 15 min and analyzed in phenol red-free medium containing FBS using a Carl Zeiss LSM800 (Carl Zeiss Microimaging GmbH, Jena, Germany).

### 2.10. Endocytosis pathway analysis

The cells were seeded (2.5 × 10^4^ cells/cm^2^) and incubated for 24 h. The cells were preincubated with inhibitors [sucrose (0.4 M), genistein (200 μM), 5-(N-ethyl-N-isopropyl)-amiloride (EIPA) (50 μM)] or at 4 °C for 30 min. Then, in the presence of each inhibitor or after culture at 4 °C, the cells were incubated with KK-(EK)_4_/PEGylated liposomes for 30 min. After incubation, the cells were washed with PBS, collected, and analyzed using a BD LSR Fortessa flow cytometry (BD Biosciences, San Jose, CA, USA).

### 2.11. In vitro luciferase expression

The cells were seeded (1.9 × 10^4^ cells/cm^2^) and incubated for 24 h. mRNA-LNPs (0.1 µg of mRNA) in serum-containing medium were added to the cells and incubated for 24 h. The cells were washed with PBS and lysed with lysis buffer. Luciferase activity in the cell lysate was measured using a luminometer (Luminescencer-PSN; ATTO, Japan). The amount of protein in the cell lysate was determined using a bicinchoninic acid (BCA) protein assay kit (Pierce).

### 2.12. Statistical analysis

ANOVA was used to analyze the statistical significance of the differences between the groups. The Tukey-Kramer test was used for multiple comparisons between all groups. Dunnett’s test was used for multiple comparisons between control and treatment groups. Differences with *p*** **<** **0.05 were considered significant.

## 3. Results

### 3.1. Synthesis of (EK)_n_-lipid derivatives

**Figure 1.**
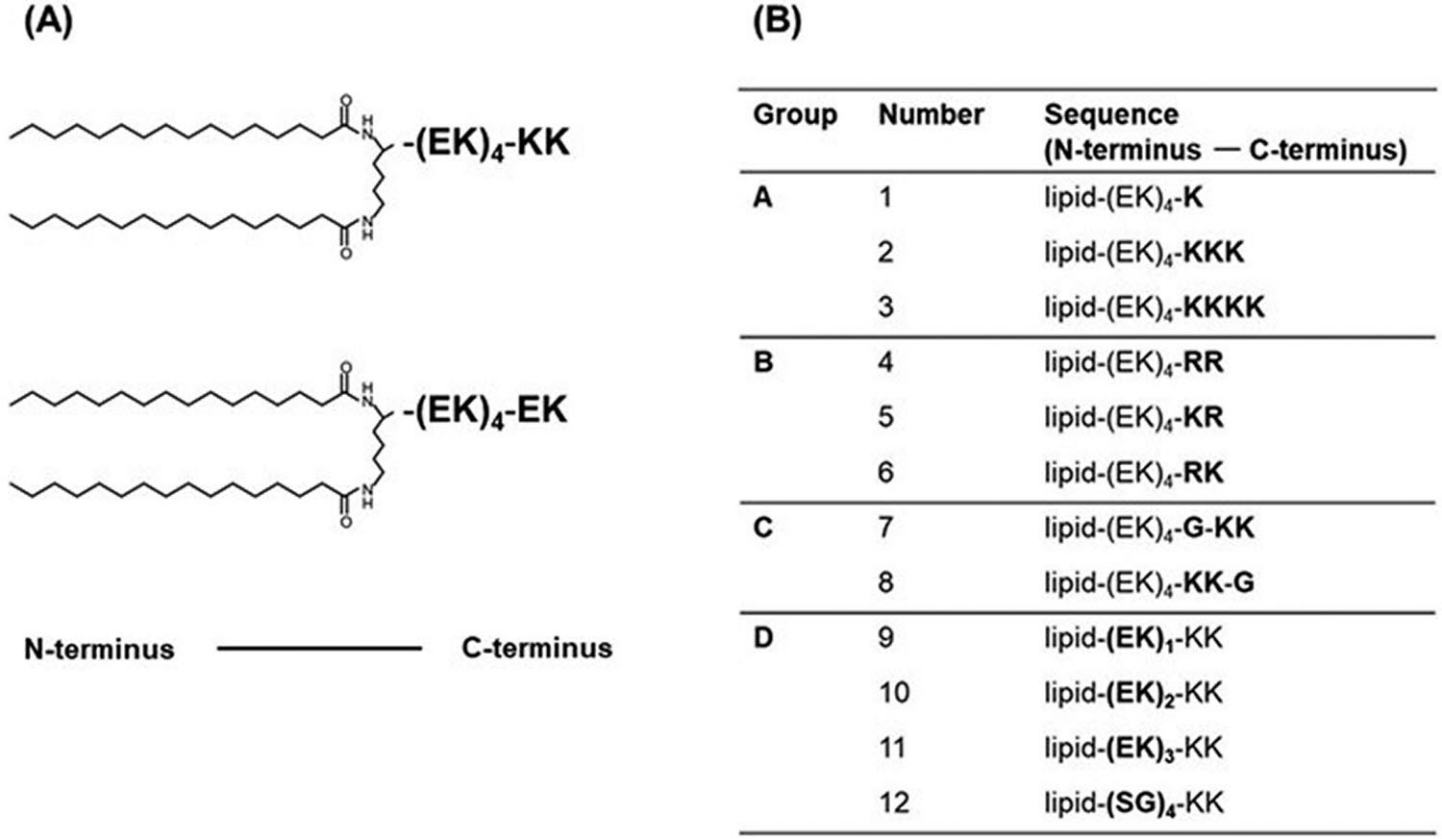
(A) Chemical structure of KK-(EK)_4_-lipid and EK-(EK)_4_-lipid. (B) Illustration of (EK)_n_-lipid derivatives.

We synthesized (EK)_n_-lipid derivatives, as shown (Figure 1). Group A: sequences with an altered number of C-terminal lysines (K); Group B: sequences with altered C-terminus lysines (K) to arginine (R); Group C: sequences with glycine (G) next to the C-terminus KK; Group D: sequences with varying lengths of lysine and glutamic acid (EK) repeats and sequences with (EK) repeats changed to (SG) repeats.

### 3.2. Cellular association of KK-(EK)_4_-lipid-modified PEGylated liposomes

**Figure 2.**
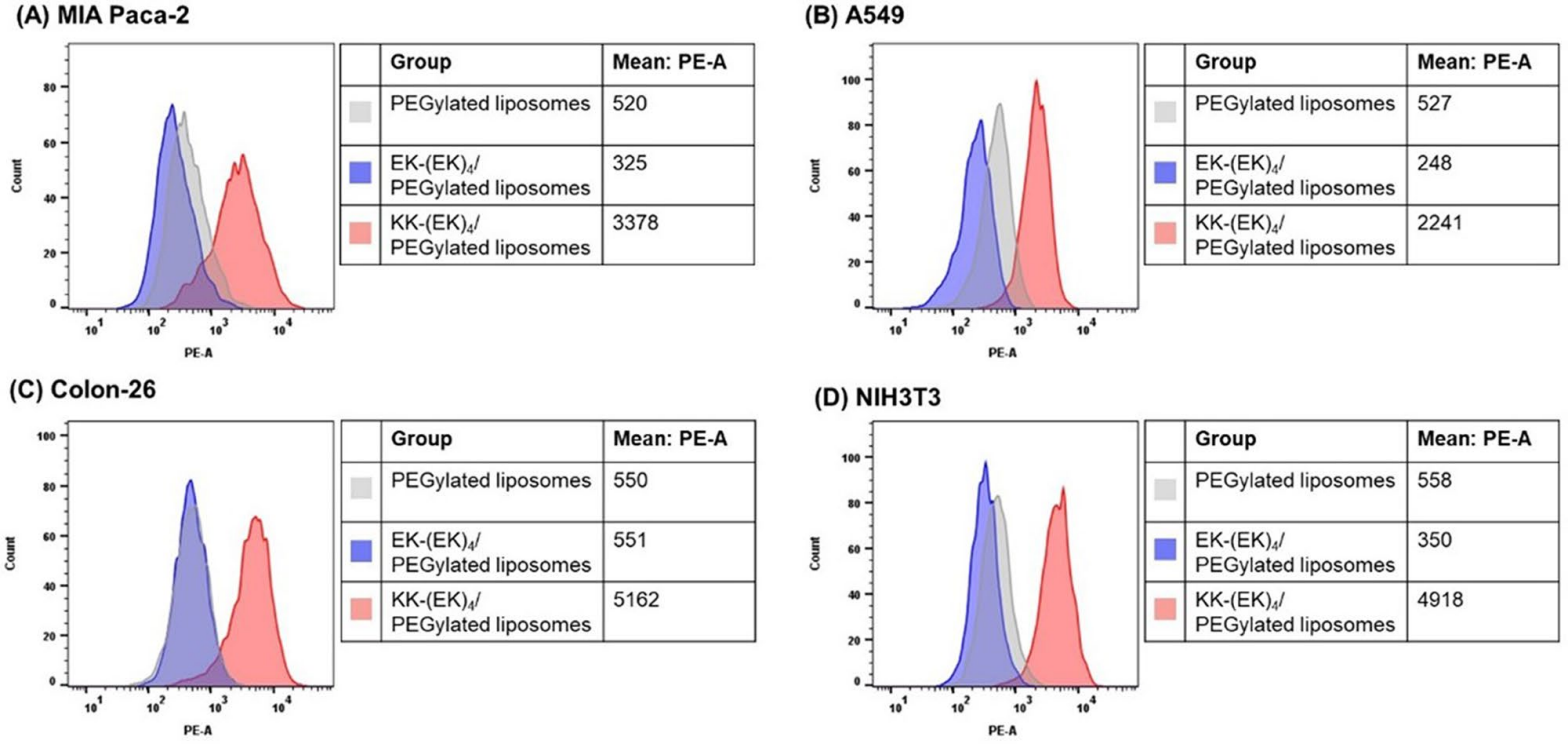
Cellular association of KK-(EK)_4_/PEGylated liposomes (at a modification amount of 6%), as analyzed using flow cytometry in (A) MIA Paca-2, (B) A549, (C) Colon-26, and (D) NIH3T3 cells. The cells were treated with 25 μM rhodamine-labeled liposomes for 3 h.

To determine the selectivity of KK-(EK)_4_/PEGylated liposomes, the cellular association of KK-(EK)_4_/PEGylated liposomes was evaluated using normal and cancer cell lines (MIA Paca-2, A549, Colon-26, and NIH3T3 cells) (Figure 2). We used uncharged EK-(EK)_4_-lipid, which changed the lysine (K) at the end of the C-terminus to glutamic acid (E), as a control for KK-(EK)_4_-lipid. KK-(EK)_4_/PEGylated liposomes showed higher cell association properties than PEGylated liposomes and EK-(EK)_4_/PEGylated liposomes in all cell lines. Moreover, analysis of the CD spectra revealed that KK-(EK)_4_ peptide formed a random structure (Figure S1).

### 3.3. Characterization of (EK)_n_-lipid derivative-modified PEGylated liposomes

We prepared (EK)_n_-lipid derivative-modified PEGylated liposomes by incubating PEGylated liposomes with 6 mol% (EK)_n_-lipid derivatives using the bulk mixing post-insertion method (Table 1). The KK-(EK)_4_/PEGylated liposomes had a particle size equivalent to that of PEGylated liposomes, with a PDI of approximately 0.1. The zeta potential was almost neutral and slightly more positive than that of the PEGylated liposomes and EK-(EK)_4_/PEGylated liposomes. In group A, there were no significant changes in the particle size and PDI with increasing number of K. The zeta potential did not change significantly for K1 and K2 compared to PEGylated liposomes but tended to increase for K3 and K4. In group B, changing K to R had almost no effect on particle size, zeta potential, or PDI, whereas KR-(EK)_4_/PEGylated liposomes showed a slightly higher PDI. In group C, the particle size and PDI tended to increase slightly in GKK-(EK)_4_/PEGylated liposomes, whereas the zeta potential tended to increase in KKG-(EK)_4_/PEGylated liposomes. In group D, slight increases in the particle size and PDI were observed for all the sequence-evaluated modified PEGylated liposomes. In addition, PEGylated liposomes modified with sequences that reduced the number of EK repeats tended to vary in their particle size.

**Table 1.** Physicochemical properties of (EK)_n_-lipid derivative-modified PEGylated liposomes. Data are presented as the mean ± SD for triplicate experiments.

	Particle size (nm)	ζ-potential (mV)	Polydispersity index (PDI)
PEGylated liposomes	86.6 ± 0.2	−0.4 ± 0.6	0.083 ± 0.024
EK-(EK)_4_/PEGylated liposomes	84.1 ± 2.1	−0.3 ± 1.0	0.132 ± 0.033
KK-(EK)_4_/PEGylated liposomes	82.8 ± 0.2	2.4 ± 1.2	0.105 ± 0.020
K-(EK)_4_/PEGylated liposomes	84.8 ± 0.8	1.5 ± 0.4	0.127 ± 0.013
KKK-(EK)_4_/PEGylated liposomes	82.6 ± 0.5	4.3 ± 0.2	0.122 ± 0.006
KKKK-(EK)_4_/PEGylated liposomes	83.9 ± 0.6	5.3 ± 1.1	0.148 ± 0.022
RR-(EK)_4_/PEGylated liposomes	83.7 ± 1.2	3.0 ± 1.7	0.111 ± 0.010
PEGylated liposomes	87.7 ± 5.4	−1.0 ± 0.5	0.156 ± 0.060
EK-(EK)_4_/PEGylated liposomes	82.0 ± 8.3	−0.5 ± 0.7	0.142 ± 0.011
KK-(EK)_4_/PEGylated liposomes	87.6 ± 2.4	0.0 ± 1.4	0.153 ± 0.023
RK-(EK)_4_/PEGylated liposomes	96.9 ± 12.9	0.5 ± 1.4	0.170 ± 0.046
KR-(EK)_4_/PEGylated liposomes	97.6 ± 7.3	0.6 ± 0.7	0.233 ± 0.078
KK-(EK)_1_/PEGylated liposomes	111.2 ± 31.5	0.6 ± 1.2	0.229 ± 0.108
KK-(EK)_2_/PEGylated liposomes	125.4 ± 55.8	0.4 ± 0.3	0.253 ± 0.174
KK-(EK)_3_/PEGylated liposomes	100.2 ± 17.2	−0.8 ± 1.0	0.207 ± 0.096
KK-(SG)_4_/PEGylated liposomes	112.3 ± 9.5	−0.2 ± 0.6	0.201 ± 0.064
PEGylated liposomes	87.2 ± 0.9	0.3 ± 0.9	0.098 ± 0.019
EK-(EK)_4_/PEGylated liposomes	87.6 ± 1.4	−0.1 ± 0.4	0.105 ± 0.016
KK-(EK)_4_/PEGylated liposomes	89.6 ± 4.0	1.7 ± 0.9	0.124 ± 0.030
KKG-(EK)_4_/PEGylated liposomes	88.0 ± 1.1	2.4 ± 1.0	0.129 ± 0.018
GKK-(EK)_4_/PEGylated liposomes	95.2 ± 15.3	1.3 ± 1.3	0.166 ± 0.080

### 3.4. Cellular association of (EK)_n_-lipid derivative-modified PEGylated liposomes

**Figure 3.**
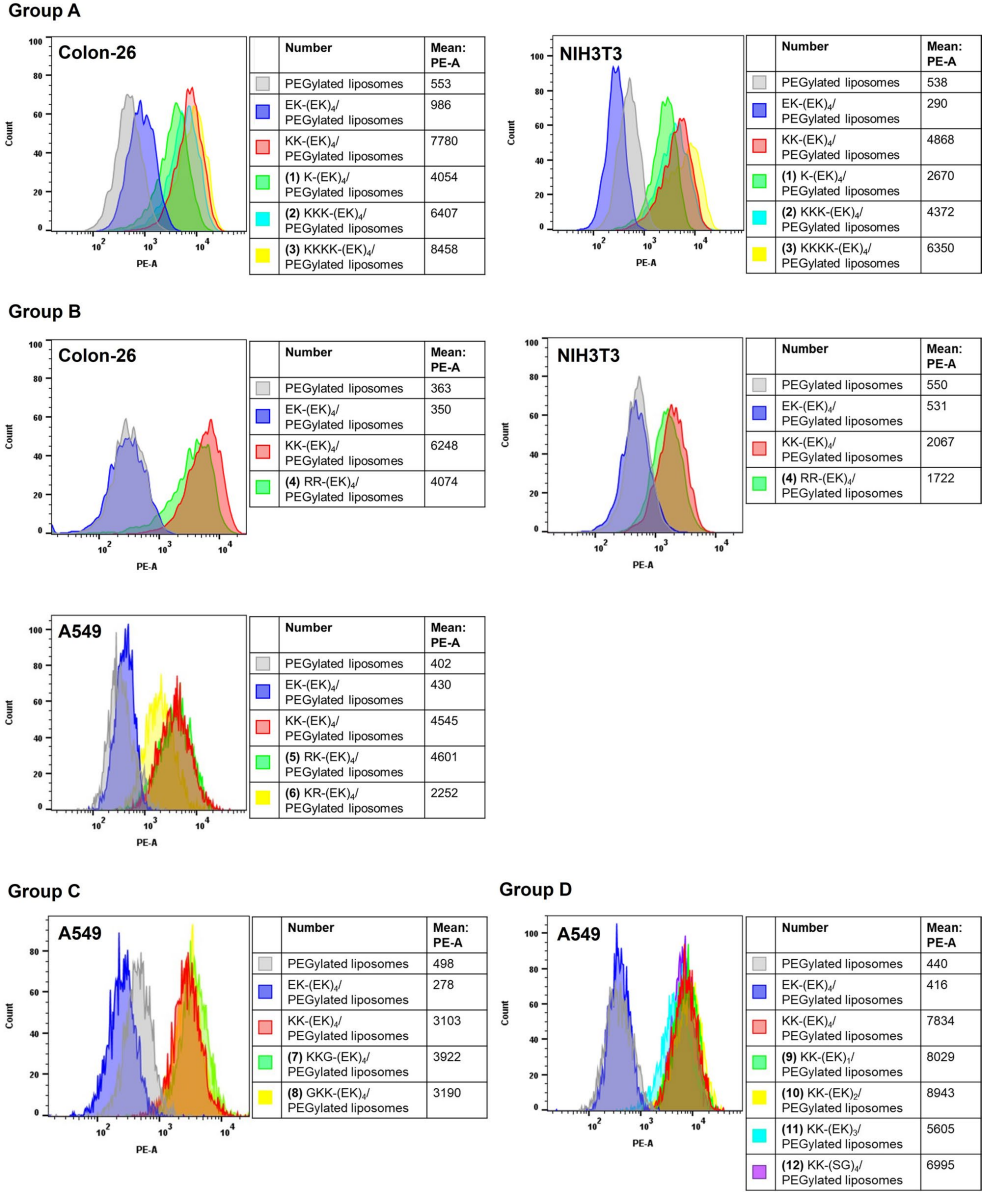
Cellular association of (EK)_n_-lipid derivatives/PEGylated liposomes (at a modification amount of 6%), as analyzed using flow cytometry. The cells were treated with 25 µM rhodamine-labeled liposomes for 3 h. The groups and numbers in the figure correspond to Figure 1(B).

Next, to investigate the effect of amino acids in KK-(EK)_4_-lipid on cellular association, we evaluated the cellular association of (EK)_n_-lipid derivative-modified PEGylated liposomes (Figure 3).

Group A: Liposomes modified with a sequence in which one K was added after (EK)_4_ (K-(EK)_4_-lipid) showed a remarkable increase in fluorescence intensity by approximately 8-fold in Colon-26 cells and 5-fold in NIH3T3 cells compared to PEGylated liposomes. However, when two or more lysines were added (KK-(EK)_4_-lipid, KKK-(EK)_4_-lipid, and KKKK-(EK)_4_-lipid), only an increase in the fluorescence intensity of the liposomes of approximately 1000 was observed for each addition of one K.

Group B: Changing the C-terminus K to one or two R did not affect the cellular association of liposomes, and the fluorescence intensity of the liposomes was higher than that of PEGylated liposomes, which was comparable to that of KK-(EK)_4_-lipid-modified liposomes. On the other hand, only KR-(EK)_4_/PEGylated liposomes showed a fluorescence intensity approximately half that of KK-(EK)_4_/PEGylated liposomes.

Group C: Both liposomes with (KKG-(EK)_4_/PEGylated liposomes) and without lysine (GKK-(EK)_4_/PEGylated liposomes) at the C-terminus showed a high fluorescence intensity equivalent to that of KK-(EK)_4_/PEGylated liposomes.

Group D: When the spacer was (EK)_1_, the fluorescence intensity of liposomes was dramatically increased by approximately 20 times compared to that of PEGylated liposomes. Moreover, longer spacers ((EK)_2_, (EK)_3_, and (EK)_4_) and changing the spacer to (SG)_4_ had little effect on the fluorescence intensity of the liposomes.

### 3.5. Intracellular distribution of KK-(EK)_4_-lipid-modified PEGylated liposomes

**Figure 4.**
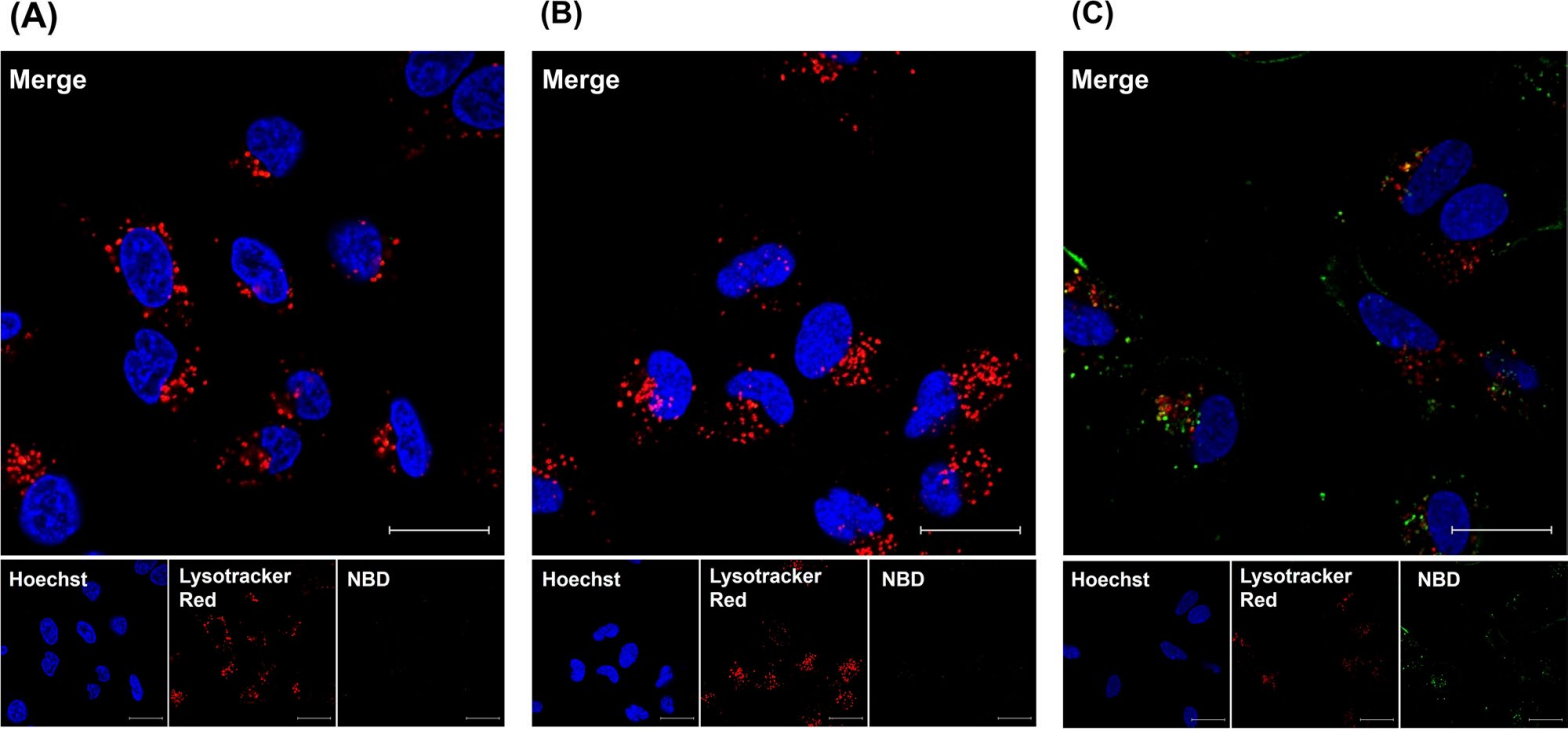
Confocal microscopy images of PEGylated liposomes (A), EK-(EK)_4_/PEGylated liposomes (B), and KK-(EK)_4_/PEGylated liposomes (C) (at a modification amount of 6%) in A549 cells. The cells were treated with 25 µM NBD-labeled liposomes for 3 h. Nuclei were stained with Hoechst 33342 (blue). Late endosomes/lysosomes were stained with LysoTracker Red (red). Liposomes have been indicated as green fluorescence. Scale bar = 20 μm.

In addition to evaluating cell association properties via flow cytometry, we evaluated the intracellular localization of KK-(EK)_4_/PEGylated liposomes in A549 cells using confocal laser microscopy (Figure 4). Strong fluorescence of liposomes was observed in cells treated with KK-(EK)_4_/PEGylated liposomes compared to cells treated with PEGylated liposomes and EK-(EK)_4_/PEGylated liposomes, consistent with the data obtained via flow cytometry. Moreover, KK-(EK)_4_/PEGylated liposomes were colocalized in late endosomes/lysosomes and in the cytoplasm.

### 3.6. Endocytosis pathway study of KK-(EK)_4_-lipid-modified PEGylated liposomes

**Figure 5.**
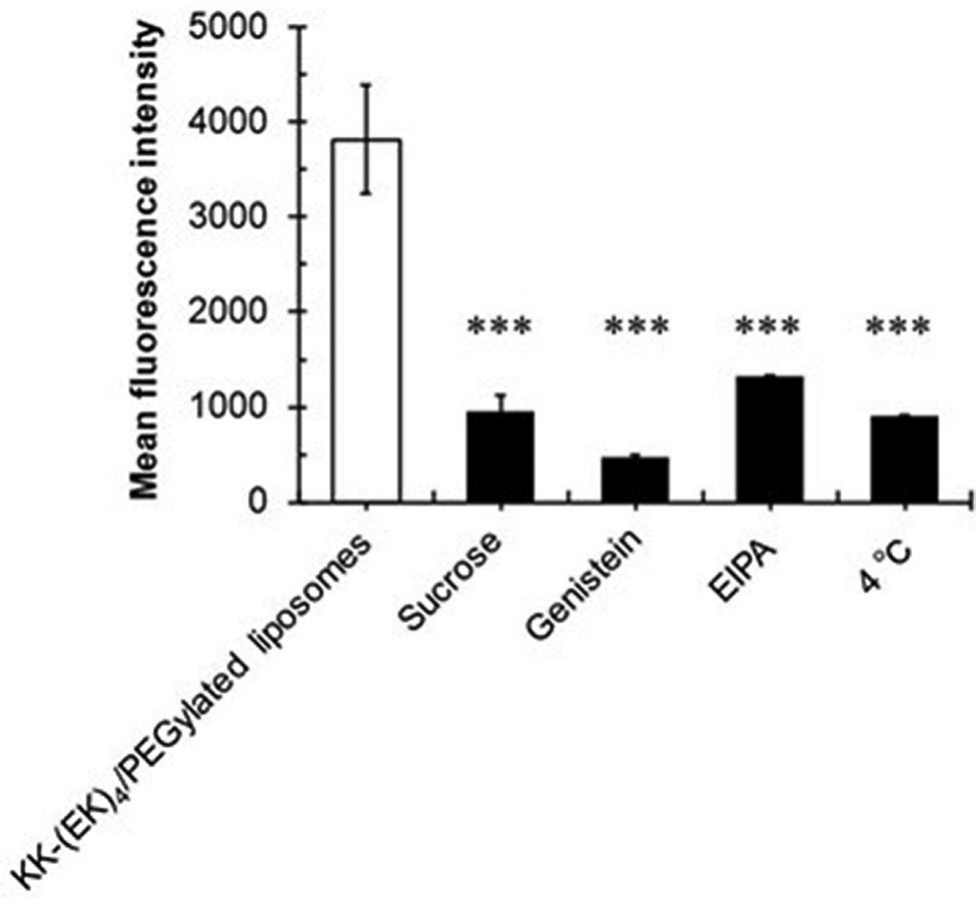
Endocytosis pathway analysis of KK-(EK)_4_/PEGylated liposomes (at a modification amount of 6%) in A549 cells. After pre-incubation with the inhibitors or condition, the cells were incubated with 25 µM rhodamine-labeled liposomes, and the fluorescence intensity of liposomes was analyzed via flowcytometry. A group treated with KK-(EK)_4_/PEGylated liposomes was used as the control. Data are presented as the mean ± SD for triplicate experiments. ***p < 0.001.

To determine the intracellular pathway, we evaluated the cellular association of KK-(EK)_4_/PEGylated liposomes in A549 cells using endocytosis inhibitors (sucrose, an inhibitor of clathrin-mediated endocytosis; genistein, an inhibitor of caveolae-mediated endocytosis; EIPA, an inhibitor of macropinocytosis; and 4 °C, used as a condition to inhibit all endocytosis) (Figure 5). After pretreatment with inhibitors or condition, 6 mol% KK-(EK)_4_/PEGylated liposomes were added, and liposome cellular association was evaluated. A significant reduction in cellular uptake was observed in pretreated cells with inhibitors or condition compared to non-pretreated cells.

### 3.7. Preparation of KK-(EK)_4_-lipid-modified PEGylated liposomes using microfluidic post-insertion method

**Figure 6.**
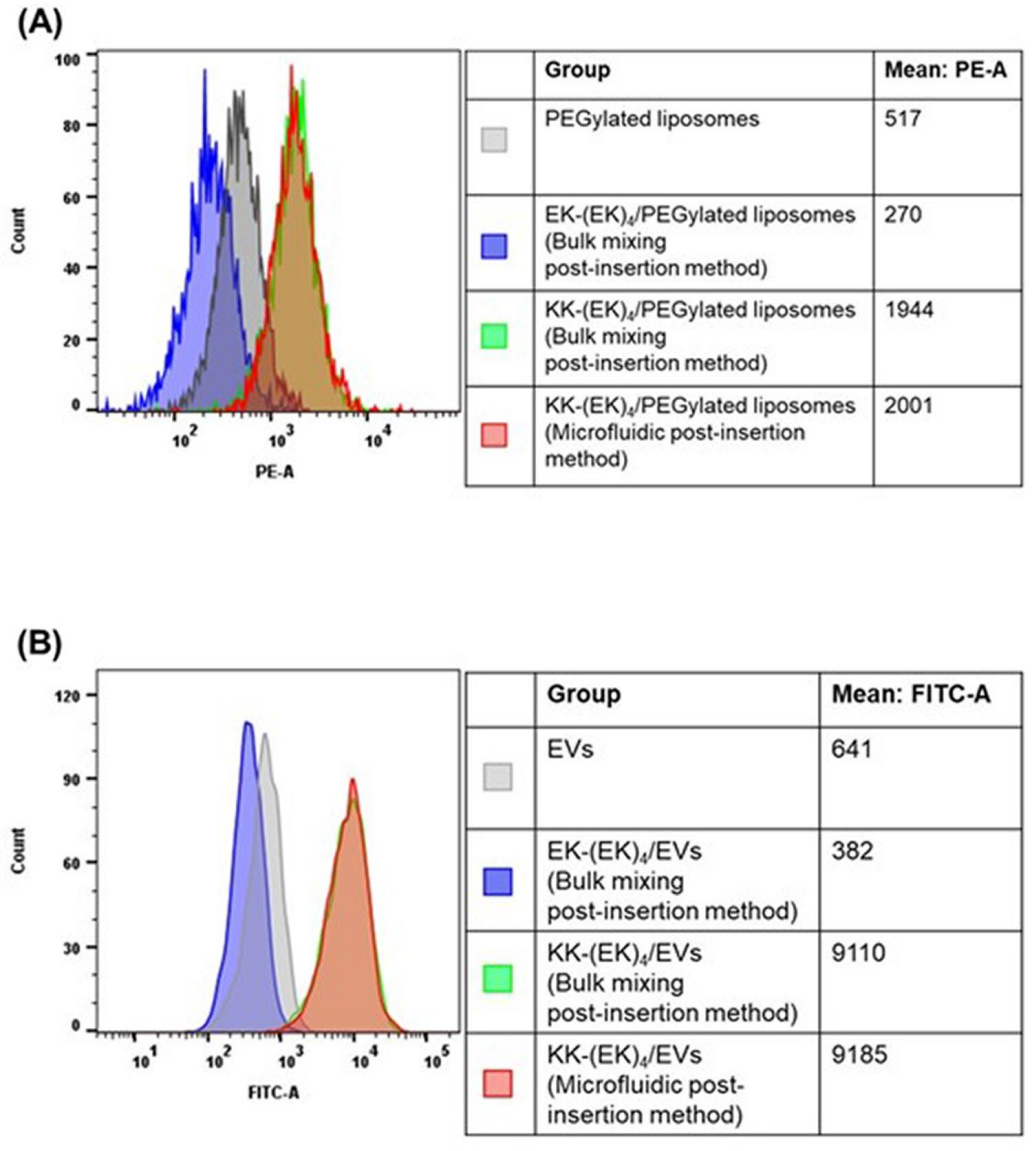
Cellular association of KK-(EK)_4_-lipid-modified nanoparticles prepared using microfluidic post-insertion method ((A) KK-(EK)_4_/PEGylated liposomes (at a modification amount of 6%) and (B) KK-(EK)_4_/EVs (protein amount of KK-(EK)_4_-lipid micelles and EVs = 1:1), as analyzed via flow cytometry in A549 cells. The cells were treated with 25 μM rhodamine-labeled liposomes or 5 µg/mL CFSE-labeled EVs for 3 h.

In the evaluation described thus far, we assessed the cellular association properties of KK-(EK)_4_/PEGylated liposomes prepared using bulk mixing post-insertion method. In the microfluidic post-insertion method, functional lipids can be modified onto liposomes by uniformly mixing the aqueous solvent and the aqueous solvent using a microfluidic device. Therefore, because KK-(EK)_4_-lipid has high dispersity in aqueous solvents, we evaluated whether KK-(EK)_4_/PEGylated liposomes can be prepared using the microfluidic post-insertion method. KK-(EK)_4_/PEGylated liposomes exhibited similar particle sizes and PDI to liposomes prepared using the bulk mixing post-insertion method, while exhibiting a slightly higher zeta potential (Table 2). Moreover, KK-(EK)_4_/PEGylated liposomes prepared using the microfluidic post-insertion method showed higher cellular association than PEGylated liposomes, which was comparable to liposomes prepared using the bulk mixing post-insertion method (Figure 6(A)). These results suggest that KK-(EK)_4_-lipid is modified on the surface of liposomes using a microfluidic device.

**Table 2.** Physicochemical properties of KK-(EK)_4_-lipid-modified PEGylated liposomes prepared using various methods. Data are presented as the mean ± SD for triplicate experiments.

	Particle size (nm)	ζ-potential (mV)	Polydispersity index (PDI)
PEGylated liposomes	83.9 ± 1.2	0.7 ± 0.8	0.109 ± 0.005
EK-(EK)_4_/PEGylated liposomes (Bulk mixing post-insertion method)	82.7 ± 0.7	−0.4 ± 1.2	0.103 ± 0.016
KK-(EK)_4_/PEGylated liposomes (Bulk mixing post-insertion method)	82.1 ± 0.9	1.2 ± 0.6	0.138 ± 0.040
KK-(EK)_4_/PEGylated liposomes (Microfluidic post-insertion method)	82.6 ± 0.8	2.5 ± 0.7	0.132 ± 0.026

### 3.8. Preparation of KK-(EK)_4_-lipid-modified EVs using microfluidic post-insertion method

On assessing the data regarding KK-(EK)_4_/PEGylated liposomes prepared using the microfluidic post-insertion method, we hypothesized that rapid mixing using a microfluidic device could also be applied to EVs with a lipid bilayer membrane similar to liposomes. KK-(EK)_4_/EVs prepared using the microfluidic post-insertion method and those prepared using the bulk mixing post-insertion method exhibited almost the same particle size and PDI as unmodified EVs, whereas the zeta potential of those prepared using the microfluidic post-insertion method was slightly higher (Table 3). Moreover, KK-(EK)_4_/EVs obtained by the bulk mixing post-insertion method and the microfluidic post-insertion method showed comparable cellular association, which was higher than that of unmodified EVs (Figure 6(B)).

**Table 3.** Physicochemical properties of KK-(EK)_4_-lipid-modified EVs. Data are presented as the mean ± SD for triplicate experiments.

	Particle size (nm)		ζ-potential (mV)	Polydispersity index (PDI)
	z-average	Number average		
EVs	213.5 ± 18.3	134.7 ± 10.6	−5.2 ± 2.4	0.205 ± 0.052
EK-(EK)_4_/EVs (Bulk mixing post-insertion method)	198.1 ± 16.7	121.7 ± 34.4	−6.8 ± 1.1	0.169 ± 0.054
KK-(EK)_4_/EVs (Bulk mixing post-insertion method)	227.5 ± 19.8	138.4 ± 14.2	−6.9 ± 2.2	0.212 ± 0.056
KK-(EK)_4_/EVs (Microfluidic post-insertion method)	230.7 ± 13.6	111.8 ± 42.4	−4.3 ± 2.5	0.217 ± 0.018

### 3.9. Preparation of KK-(EK)_4_-lipid-modified mRNA-LNPs using self-assembly method in microfluidic device

**Figure 7.**
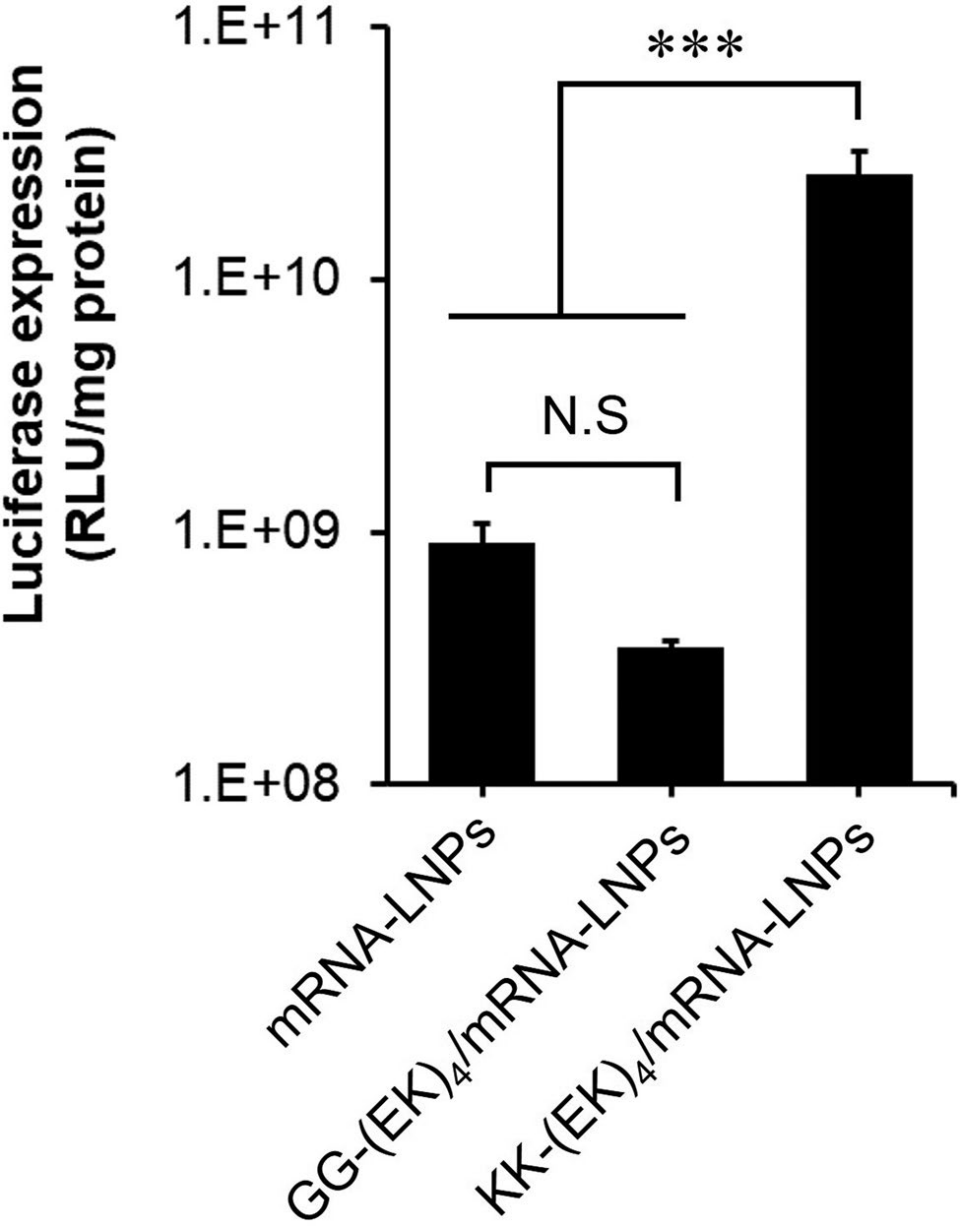
*In vitro* luciferase expression of KK-(EK)_4_/mRNA-LNPs in A549 cells. The cells were treated with mRNA-LNPs (0.1 μg as mRNA) for 24 h. Data are presented as the mean ± SD for triplicate experiments. ***p < 0.001.

In the evaluations described thus far, we prepared KK-(EK)_4_-lipid-modified nanoparticles by mixing them in aqueous solvents. On the other hand, since KK-(EK)_4_-lipid is also highly soluble in ethanol, we performed a one-step modification of mRNA-LNPs by self-assembly using a microfluidic device. To prepare KK-(EK)_4_-lipid-modified mRNA-LNPs, KK-(EK)_4_-lipid was dissolved in ethanol together with lipids and mixed with an aqueous solvent containing mRNA on a microfluidic device. Since EK-(EK)_4_-lipid, which has been used as a control, did not show solubility in ethanol, we synthesized GG-(EK)_4_-lipid as a control. The prepared KK-(EK)_4_/mRNA-LNPs exhibited particle sizes similar to those of unmodified mRNA-LNPs, while their PDI and zeta potential tended to increase (Table 4). Moreover, they showed comparable encapsulation efficacy (EE) to that of unmodified mRNA-LNPs and GG-(EK)_4_-lipid-modified mRNA-LNPs. In the in vitro luciferase assay, KK-(EK)_4_/mRNA-LNPs showed higher luciferase expression compared to unmodified mRNA-LNPs and GG-(EK)_4_/mRNA-LNPs (Figure 7).

**Table 4.** Physicochemical properties of KK-(EK)_4_-lipid-modified mRNA-LNPs. Data are presented as the mean ± SD for triplicate experiments.

	Particle size (nm)	ζ-potential (mV)	Polydispersity index (PDI)	Encapsulation efficiency (%)
mRNA-LNPs	95.5 ± 3.3	−1.4 ± 1.8	0.048 ± 0.018	89.6 ± 2.1
GG-(EK)_4_/mRNA-LNPs	92.1 ± 3.5	−1.4 ± 1.9	0.111 ± 0.027	88.8 ± 1.2
KK-(EK)_4_/mRNA-LNPs	99.9 ± 9.9	0.7 ± 3.3	0.161 ± 0.045	88.3 ± 1.9

## 4. Discussion

In this study, we synthesized (EK)_n_-lipid derivatives and evaluated the cellular association properties of PEGylated liposomes to reveal the cellular association mechanism of KK-(EK)_4_-lipid. Furthermore, we applied KK-(EK)_4_-lipid to EVs and mRNA-LNPs and evaluated whether KK-(EK)_4_-lipid could be a novel CPP-modified lipid for functionalized nanoparticles.

First, we synthesized various (EK)_n_-lipid derivatives to analyze the effect of the amino acid sequence of KK-(EK)_4_-lipid on cellular association and prepared PEGylated liposomes modified with (EK)_n_-lipid derivatives using the bulk mixing post-insertion method. Most liposomes showed a particle size of approximately 100 nm and a neutral charge, whereas KK-(EK)_1_/PEGylated liposomes and KK-(EK)_2_/PEGylated liposomes with fewer EK repeats showed larger sizes and PDI (Table 1). This result might be due to the stabilization of the interface of liposomes by increasing the number of EK repeats because the repeat sequence of EK is composed of zwitterions and has been reported to form a hydrated layer similar to PEG (Nowinski et al., 2012).

In the evaluation of the cellular association of liposomes, KK-(EK)_4_/PEGylated liposomes showed higher cellular association than EK-(EK)_4_/PEGylated liposomes (Figure 2). Therefore, substitution of the C-terminus E with K might be the key for the cellular association of KK-(EK)_4_/PEGylated liposomes. In addition, enhanced cellular association was observed not only in cancer cells, but also in non-cancer cells. This result suggests that KK-(EK)_4_/PEGylated liposomes interact nonspecifically with the cell membrane.

Next, we investigated the effect of the number of C-termini on cellular associations. When only one lysine residue was added to the C-terminus of (EK)_4_ (K-(EK)_4_/PEGylated liposomes), the cellular association of the liposomes dramatically increased compared to that of completely neutral EK repeats (EK-(EK)_4_/PEGylated liposomes) (Figure 3, Group A). Furthermore, we substituted K with R (Figure 3, Group B) because previous reports have shown that the formation of hydrogen bonds between arginine residues and the cell membrane plays an important role in the cellular uptake of CPPs (Rothbard et al., 2004). However, the substitution of K with R had little effect on the cellular association of liposomes. These results suggest that one or more K or R residues adjacent to the spacer sequence are essential for the drastic enhancement of cellular association.

We inserted G into the C-terminus of KK-(EK)_4_/PEGylated liposomes to mask cationic residues (GKK-(EK)_4_/PEGylated liposomes) (Figure 3, Group C) because we assumed that the terminal KK interacts with the anionic cellular membrane. However, masking the KK-(EK)_4_-lipid by introducing a single G residue at the tip of KK suggests that this does not affect the interaction between KK-(EK)_4_/PEGylated liposomes and the cell membrane.

Based on our previous report that the length and structure of the spacer in functional lipids are important factors in the cellular association of PEGylated liposomes (Suga et al., 2017, 2018), we changed (EK)_4_ to (EK)_1_, (EK)_2_, (EK)_3_ (Figure 3, Group D) and investigated the secondary structures of KK-(EK)_4_ peptide using CD spectra. Moreover, we changed (EK)_4_ to (SG)_4_ to evaluate whether the cellular association was specific for EK repeat sequence (Figure 3, Group D). KK-(EK)_4_ peptide displayed a random structure (Figure S1). In addition, liposomes modified with each sequence showed higher cell association than PEGylated liposomes. Previous reports have shown that the thickness PEG layer is approximately 3.5 nm and the length per amino acid residue is 0.37 nm (Idiris et al., 2000; Garbuzenko et al., 2005). Based on these reports, in sequences that have shorter repeats of (EK)_n_ ((EK)_1_, (EK)_2_, and (EK)_3_), the C-terminus lysine (K) might be masked by the PEG layer. Therefore, although the present analysis is only phenomenological and unresolved, the cellular association properties of (EK)_n_-lipid derivatives might be attributable not only to cationic amino acid residues but also to other factors.

We evaluated the cellular uptake mechanism of the KK-(EK)_4_/PEGylated liposomes in A549 cells. Evaluation of the cellular association of KK-(EK)_4_/PEGylated liposomes using endocytosis inhibitors (Figure 5) suggested that KK-(EK)_4_/PEGylated liposomes were translocated into cells through endocytosis, including clathrin-mediated endocytosis, caveolae-mediated endocytosis, and macropinocytosis. In addition, during the evaluation of intracellular distribution by confocal laser microscopy (Figure 4), fluorescence of KK-(EK)_4_/PEGylated liposomes was observed in the cytoplasm. These results suggest that KK-(EK)_4_/PEGylated liposomes are taken up by A549 cells via the endocytic pathway and then translocates to and escapes from endosomes. We assume that one of the cellular uptake pathways of KK-(EK)_4_/PEGylated liposomes is macropinocytosis, which is involved in the translocation of CPP-modified nanoparticles to the cytoplasm, consistent with a previous report on octaarginine, a CPP peptide modification (Khalil et al., 2006).

When mixing in the microchannel using a microfluidic device, it is possible to uniformly mix the solvents. Since KK-(EK)_4_-lipid has high dispersity in water and solubility in ethanol, we prepared KK-(EK)_4_-lipid-modified nanoparticles using a microfluidic device. First, by mixing aqueous solvents, KK-(EK)_4_-lipid was modified into PEGylated liposomes. KK-(EK)_4_/PEGylated liposomes showed almost the same physicochemical properties (particle size, PDI, and zeta potential) (Table 2) and higher cellular association than PEGylated liposomes as liposomes prepared using the conventional method (Figure 6(A)). These results suggest that KK-(EK)_4_-lipid can be modified on the PEGylated liposome surface using a microfluidic device.

The bulk mixing post-insertion method has been used to functionalize EVs and requires mixing of EVs and micelles for a long period of time (30 min to 3 h under heat) (Choi et al., 2019; Lin et al., 2019; Zhu et al., 2019). However, since liposomes could be modified with KK-(EK)_4_-lipid by short-term contact using a microfluidic device, we hypothesized that mixing using a microfluidic device was applicable to the preparation of KK-(EK)_4_/EVs. When EVs were modified with KK-(EK)_4_-lipid, the modification of KK-(EK)_4_-lipid did not significantly change the physicochemical properties of unmodified EVs in either the microfluidic post-insertion method or the bulk mixing post-insertion method (Table 3). This result might be because KK-(EK)_4_-lipid is a functional lipid with an almost neutral charge that can reduce interactions with EVs that have a large negative charge. Moreover, it has been reported that the EK repeat sequence prevents the adsorption of proteins similar to PEG (Nowinski et al., 2012), and thus, KK-(EK)_4_-lipid could be modified while preventing EVs aggregation. Furthermore, KK-(EK)_4_/EVs prepared using both methods exhibited higher cell-association properties than unmodified EVs (Figure 6(B)). These results suggest that the microfluidic post-insertion method can be applied to EVs and liposomes, taking advantage of the properties of KK-(EK)_4_-lipids, which are highly dispersible in water, to impart and improve cell-association properties while maintaining the original physicochemical properties of EVs.

We prepared KK-(EK)_4_-lipid-modified mRNA-LNPs by a self-assembly method using a microfluidic device. In the evaluation of physicochemical properties, modification of GG-(EK)_4_-lipid and KK-(EK)_4_-lipid did not affect the particle size of mRNA-LNPs but increased the PDI (Table 4). On the other hand, KK-(EK)_4_/mRNA-LNPs and GG-(EK)_4_/mRNA-LNPs showed encapsulation efficiencies comparable to those of unmodified mRNA-LNPs, and these encapsulation efficiencies were consistent with a previous report on the modification of peptide-modified lipids into mRNA-LNPs by self-assembly using a microfluidic device (Qin et al., 2022). In addition, in the evaluation of luciferase expression in A549 cells, KK-(EK)_4_/mRNA-LNPs showed higher expression than unmodified mRNA-LNPs and GG-(EK)_4_/mRNA-LNPs (Figure 7). This result suggests that the C-terminal K of KK-(EK)_4_-lipid contributes to the delivery of mRNA into the cytoplasm, consistent with the intracellular distribution of KK-(EK)_4_/PEGylated liposomes (Figure 4). Therefore, KK-(EK)_4_-lipid might be used to improve the cellular association and subsequent intracellular delivery of mRNA-LNPs.

## 5. Conclusion

In this study, we identified the cationic amino acid K after the (EK)_n_ sequence that contributes to the cellular association of novel KK-(EK)_4_-lipid by synthesizing and evaluating various amino acid sequences based on the sequences of KK-(EK)_4_-lipid. A cellular association study revealed that KK-(EK)_4_-lipid-modified liposomes are taken up by A549 cells through endocytosis, following which they escape from endosomes and translocate into the cytoplasm, despite KK-(EK)_4_-lipid having a near-neutral charge. In addition, since KK-(EK)_4_-lipid has high dispersity and solubility in solvents, we applied KK-(EK)_4_-lipid to the functionalization of EVs and mRNA-LNPs using a microfluidic device. Moreover, in the evaluation of mRNA-LNPs, KK-(EK)_4_-lipid-modified mRNA-LNPs showed high luciferase expression, supporting the above liposome data, indicating that KK-(EK)_4_-lipid contributes to the delivery of mRNA to the cytoplasm. Although further studies on the mechanism of cellular association of KK-(EK)_4_-lipid and efficacy in vivo are required, these findings about KK-(EK)_4_-lipid and its derivatives would be valuable for the intracellular delivery of various nanocarriers with lipid membranes.

## Supplementary Material

Supplemental Material
