# Supplementary material for: Investigation of enhanced intracellular delivery of nanomaterials modified with novel cell-penetrating zwitterionic peptide-lipid derivatives
Source: Drug Deliv. 2023 Mar 25;30(1):2191891. doi: 10.1080/10717544.2023.2191891 (PMC10044157; doi:10.1080/10717544.2023.2191891)
Supplement: Supplemental Material [file IDRD_A_2191891_SM2004.docx]

**Supporting information**

***Drug Delivery***

Research Article

**Investigation of enhanced intracellular delivery of nanomaterials modified with novel cell-penetrating zwitterionic peptide-lipid derivatives**

Yuri Sugimoto,^a,b,†^ Tadaharu Suga,^a,†^ Mizuki Umino,^a^ Asako Yamayoshi,^b^ Hidefumi Mukai,^a,c^ and Shigeru Kawakami^*,a^

*^a^Department of Pharmaceutical Informatics, Graduate School of Biomedical Sciences, Nagasaki University, 1-7-1 Sakamoto-machi, Nagasaki 852-8588, Japan*

^b^*Department of Chemistry of Functional Molecules, Graduate School of Biomedical Sciences*, *Nagasaki University*, *1-14 Bunkyo-machi, Nagasaki 852-8521, Japan*

^c^*Laboratory for Molecular Delivery and Imaging Technology, RIKEN Center for Biosystems Dynamics Research, Kobe, Japan*

^†^These authors contributed equally to this work

^*^ Correspondence e-mail: skawakam@nagasaki-u.ac.jp

**Correspondence: Shigeru Kawakami, Ph.D.**

Graduate School of Biomedical Sciences, Nagasaki University, 1-7-1 Sakamoto, Nagasaki-shi, Nagasaki 852-8588, Japan

Tel/fax +81 95 819 8563

Email skawakam@nagasaki-u.ac.jp

1. **Methods**

***1.1　Circular Dichroism (CD) Spectrum Analysis***

CD spectra were measured at room temperature in a 1.0 mm path length cell using a JASCO J-725N spectropolarimeter (JASCO). Data are expressed as the residue molar ellipticity [θ]_R_ (deg·cm^2^·dmol^–1^). Phosphate buffered saline (PBS, pH 7.4) and 50/50 mixture of acetonitrile and water were used as solvents.

**Supporting Figures**


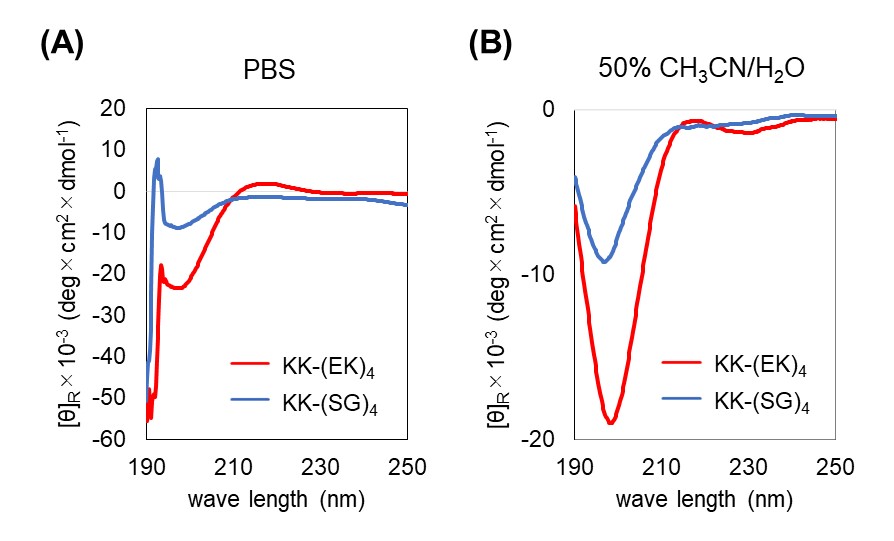


Figure S1. CD spectra of KK-(EK)_4_ and KK-(SG)_4_ peptides in (A) PBS (pH = 7.4), and (B) 50% CH_3_CN/H_2_O.
